# Decreased MicroRNA-125a-3p Contributes to Upregulation of p38 MAPK in Rat Trigeminal Ganglions with Orofacial Inflammatory Pain

**DOI:** 10.1371/journal.pone.0111594

**Published:** 2014-11-07

**Authors:** Yingchun Dong, Pengfei Li, Yanhong Ni, Junjie Zhao, Zhiqiang Liu

**Affiliations:** 1 Department of Anesthesiology, Institute and Hospital of Stomatology, Nanjing University Medical School, Nanjing, China; 2 Department of Laboratory, Jiangsu Province Hospital of Traditional Chinese Medicine, Affiliated Hospital of Nanjing University of Traditional Chinese Medicine, Nanjing, China; 3 Central Laboratory, Institute and Hospital of Stomatology, Nanjing University Medical School, Nanjing, China; 4 Department of Periodontics, Institute and Hospital of Stomatology, Nanjing University Medical School, Nanjing, China; 5 Department of Lymphoma and Myeloma, the University of Texas MD Anderson Cancer Center, Houston, United States of America; IIBB/CSIC/IDIBAPS, Spain

## Abstract

Orofacial inflammatory pain is a difficult clinical problem, and the specific molecular mechanisms for this pain remain largely unexplained. The present study aimed to determine the differential expression of microRNAs (miRNAs) and disclose the underlying role of miR-125a-3p in orofacial inflammatory pain induced by complete Freund's adjuvant (CFA). Thirty-two differentially expressed miRNAs were first screened using a microarray chip in ipsilateral trigeminal ganglions (TGs) following CFA injection into the orofacial skin innervated by trigeminal nerve, and a portion of them, including miR-23a*, -24-2*, -26a, -92a, -125a-3p, -183 and -299 were subsequently selected and validated by qPCR. The target genes were predicted based on the miRWalk website and were further analyzed by gene ontology (GO). Further studies revealed miR-125a-3p expression was down-regulated, whereas both the expression of p38 MAPK (mitogen-activated protein kinase) alpha and CGRP (calcitonin gene-related peptide) were up-regulated in ipsilateral TGs at different time points after CFA injection compared with control. Furthermore, mechanistic study revealed that miR-125a-3p negatively regulates p38 alpha gene expression and is positively correlated with the head withdrawal threshold reflecting pain. Luciferase assay showed that binding of miR-125a-3p to the 3′UTR of p38 alpha gene suppressed the transcriptional activity, and overexpression of miR-125a-3p significantly inhibited the p38 alpha mRNA level in ND8/34 cells. Taken together, our results show that miR-125a-3p is negatively correlated with the development and maintenance of orofacial inflammatory pain via regulating p38 MAPK.

## Introduction

Chronic inflammatory pain of orofacial region has been a difficult clinical problem due to complicated mechanisms. Currently, reported mechanisms describing the pain include release of inflammatory mediators and neuropeptides [1], [2], aberrant expression of pain-relevant genes [3], [4] and peripheral and central sensitization [5], [6], ect. The trigeminal ganglion (TG) is a crucial site for pain transmission and pain modulation from the peripheral to central nerve system in oral maxillofacial region [7]. Therefore, a better understanding of the cellular and molecular mechanisms underlying the pain transmission in the TG is critical for a better pain control.

MicroRNAs (miRNAs) represent a class of small non-protein-coding RNAs that regulate gene expression by binding to the 3′ UTRs (untranslated regions) of their target mRNAs and induce either translation repression or RNA degradation. It is predicted that nearly one-third of protein-encoding genes are regulated by miRNAs. Functions of miRNAs vary in diverse biological and pathological processes, including development, differentiation, regeneration, and oncogenesis [8], [9]. Furthermore, emerging evidences suggest the potential involvement of miRNAs in the development and/or maintenance of inflammatory, cancer and neuropathic pain [10]–[15].

Bali *et al*. [11] explored the roles of several miRNAs such as miR-370-3p, -1a-3p and -544-3p in a mouse model of metastatic bone-cancer pain and found these miRNAs might be important modulators reflecting cancer-associated hypersensitivity. Li *et al*. [13] reported spinal miR-146a and -183 cluster expression in osteoarthritic pain of knee joints. Besides, other studies reported the differential expressions of miRNAs in animal models with chronic neuropathic pain induced by lumbar nerve injury at peripheral and central nerve system level [10], [12], [15]. However, there were limited studies reported in the orofacial region. Poh *et al*. [14] reported the miRNA expression profile in prefrontal cortex after facial carrageenan injection and Bai *et al*. [16] detected the expression of several neuron-specific mature miRNAs including miR-10a, -29a, -98a and -134a in TGs of rats with facial complete Freund's adjuvant (CFA) injection. The roles of other miRNAs in orofacial inflammatory pain were not reported.

The present study first aims to determine the differential expression of miRNAs using miRNA microarray in rat TGs after CFA injection. We detected the down-regulation of miR-125a-3p and up-regulation of p38 MAPK (mitogen-activated protein kinase) and CGRP (calcitonin gene-related peptide) following the orofacial inflammatory pain. MiR-125 was reported to be related to cell stress, inflammation reaction and pain, including ultraviolet radiation stress, rheumatoid arthritis, virus or bacteria-induced neuroinflammation [17]–[19]. Some of these pathophysiological processes are related to p38 MAPK and CGRP. p38 MAPK, also called p38 alpha or MAPK14, was involved in peripheral and central sensitization and participated in the generation and maintenance of inflammatory, neuropathic pain of spinal cord or orofacial pain [20]–[22]. CGRP was involved in the regulation of inflammatory proteins and gene expressions as the downstream of p38 MAPK signaling and was found at high levels in association with the severity of joint pain of the temporomandibular disorders [23]–[25]. Based on these findings, we focused on miR-125a-3p and p38 MAPK, and hypothesized that miR-125a-3p could control CFA-induced orofacial inflammatory pain in TGs by regulating the activation of p38 MAPK signal pathway.

## Materials and Methods

### Animal Model of Orofacial Inflammatory Pain and Behavioral Tests

The experiment was approved by the Ethics Committee of Nanjing University and was performed in accordance with its guidelines. Male Sprague–Dawley rats, weighing 200–250 g, were included in the study. They were housed under controlled temperature and 12 h light/dark cycle for at least 6 days before any procedure. Food and water were provided ad libitum.

The rats received CFA or normal saline injection under general anesthesia with intraperitoneal injection of pentobarbital sodium (50 mg/kg). Briefly, 3×50 µL CFA (oil∶saline  = 1∶1, Sigma, St. Louis, MO) were injected subcutaneously into three skin sites on one side of the face, roughly the areas innervated by the ophthalmic, maxillary and mandibular division of the ipsilateral trigeminal nerve. Rats following normal saline injection were used as controls. After rapid decapitation under pentobarbital sodium anesthesia (50 mg/kg), the TGs were quickly removed, rinsed using normal saline and then immediately into liquid nitrogen for RNA extraction.

Behavioral tests were evaluated by the head withdrawal thresholds inversely related to pain. An electronic von Frey anesthesiometer (IITC Life Science, CA, USA) was used at different time points after CFA or normal saline injection as described previously [26], [27]. Briefly, the experimenter's hand provided a comfortable “nest” for the rat. When the rats were accommodated and stayed in position, the electronic von Frey rigid tips were probed against the inflamed skin of the maxillary whisker pad where CFA or normal saline were injected. The force amount in grams required to elicit the pain response were recorded five times at 1-min intervals. The average of the five values was used as the head withdrawal threshold. Head withdrawal or scratching the facial regions upon von Frey filament applications was considered as a positive pain response.

### MiRNA Expression Profiling

For miRNA expression profiling, eight rats were randomly divided into CFA group and control group (normal saline group) with 4 rats in each group. They were decapitated under pentobarbital sodium (50 mg/kg) anesthesia 3 days after CFA or normal saline injection. The injection-ipsilateral TGs were removed quickly and immediately frozen in liquid nitrogen for miRNA assay.

The total RNA was extracted using Trizol reagent (Invitrogen) and miRNeasy mini kit (Qiagen), according to the manufacturer's instructions. After quantity and quality measurement, the samples were labeled using the Hy3/Hy5 power labeling kit and hybridized on the LNA Array (v.18.0; Exiqon A/S). The GenePix Pro 6.0 software (Axon) was used for grid alignment and data extraction. Expressed data were normalized using the median normalization and the significant differentially expressed miRNAs were identified through Volcano Plot filtering. The threshold we used to screen the up- or down-regulated miRNAs was fold change ≥1.5, *p*-value ≤0.05, and FDR ≤0.05. Finally, hierarchical clustering was performed to display differentially expressed miRNAs profile between the two groups.

### MiRNA Reverse Transcription and Quantitative Real-time RT-PCR (qPCR) Analysis

MiRNAs out of the up- or down-regulated miRNAs screened using miRNA microarray were selected and further validated using qPCR if they have a more than six-time and significant change (*p*<0.01), or are detectable in TGs, or reported to be related to inflammation and pain processions by other studies. We expanded the number of cases and used 16 TGs from 16 different rats each group at 3 days after CFA or normal saline injection to further detect these differentially expressed miRNAs, including miR-23a*, -24-2*, -26a, -92a, -125a-3p, -183 and -299.

Briefly, after the extraction of total RNA, cDNA was synthesized from 1 µg of total RNA using reverse primer and reverse transcriptase (Toyobo, Osaka, Japan). qPCR for miRNAs and U6 snRNA (internal control) was performed using the forward primer, URP (universal reporter primer) and SYBR green qPCR kit (Roche, Basel, Switzerland). All experiments were run in triplicate. All primers synthesized by Invitrogen were listed in Table 1.

**Table 1:** MiRNA and mRNA primer information.

MiRNAs or mRNAs	Forward primer (5′-3′)	Reverse primer (5′-3′)
miR-23a*	ACACTCCAGCTGGGGGGGTTCCTGGGGATG	CTCAACTGGTGTCGTGGAGTCGGCAATTCAGTTGAGAAATCCCA
miR-24-2*	ACACTCCAGCTGGGGTGCCTACTGAGCTGA	CTCAACTGGTGTCGTGGAGTCGGCAATTCAGTTGAGCTGTTCCT
**miR-26a**	ACACTCCAGCTGGGTTCAAGTAATCCAGGA	CTCAACTGGTGTCGTGGAGTCGGCAATTCAGTTGAGAGCCTATC
**miR-92a**	ACACTCCAGCTGGGTATTGCACTTGTCCC	CTCAACTGGTGTCGTGGAGTCGGCAATTCAGTTGAGCAGGCCGG
**miR-125a-3p**	ACACTCCAGCTGGGACAGGTGAGGTTCTTG	CTCAACTGGTGTCGTGGAGTCGGCAATTCAGTTGAGGGCTCCCA
**miR-183**	ACACTCCAGCTGGGTATGGCACTGGTAGAA	CTCAACTGGTGTCGTGGAGTCGGCAATTCAGTTGAGAGTGAATT
**miR-299**	ACACTCCAGCTGGGTGGTTTACCGTCCCAC	CTCAACTGGTGTCGTGGAGTCGGCAATTCAGTTGAGATGTATGT
**U6snRNA**	GCTTCGGCAGCACATATACTAAAAT	CGCTTCACGAATTTGCGTGTCAT
**URP**	TGGTGTCGTGGAGTCG	
**p38 MAPK**	CGAAATGACCGGCTACGTGG	GACTTCATCGTAGGTCAGGC
**CGRP**	GTGTCACTGCCCAGAAGAGATC	CAAAGTTGTCCTTCACCACACC
**GAPDH**	CCAGGTGGTCTCCTCTGACTT	GTTGCTGTAGCCAAATTCGTT

### MiRNA Target Prediction and gene ontology (GO) Analysis

The target genes of miR-23a*, -24-2*, -26a, -92a, -125a-3p, -183 and -299 were predicted based on the use of some computer-based programs, including miRWalk (http://www.umm.uni-heidelberg.de/apps/zmf/mirwalk/), Targetscan (http://www.targetscan.org/), Pictar (http://www.pictar.org/), and miRBase Targets (http://microrna.sanger.ac.uk). GO analysis was performed via the GO website (http://www.geneontology.org/) to obtain more information about these predicted target genes, including the common and specific biological processes.

### Expression of miR-125a-3p, p38 MAPK and CGRP mRNA in Rat TGs

Forty rats were sacrificed under pentobarbital sodium (50mg/kg) anesthesia at eight different time points (without injection, 12 h, 1 d, 3 d, 5 d, 7 d, 14 d and 28 d after CFA injection) with 5 cases each time point. The ipsilateral TGs at injection side were collected for the detection of p38 alpha and CGRP mRNA expression. GAPDH was used as the internal reference. The sequences of forward and reverse primers are listed in Table 1. The relative expression of miR-125a-3p at different time points was detected as mentioned above.

### Cell cultures

The mouse neuroblastoma and rat neuron hybrid ND8/34 cell line was purchased from Sigma-Aldrich (St. Louis, MO, USA). They were cultured in DMEM medium supplemented with 10% fetal bovine serum (FBS) containing 100 U/mL of penicillin and streptomycin at 37°C in an atmosphere of 5% CO_2_.

### Changes of p38 MAPK mRNA expression following over-expression of miR-125a-3p in ND8/34 cells

ND8/34 cells were transfected with 0, 25, 50 nM of miR-125a-3p mimic or inhibitor (Life Technolgoy, NY, USA) using Lipofectamine 2000 transfection reagent (Invitrogen) according to the manufacturer's instructions. The negative control samples were treated with a non-target control mimic. Cells were harvested at 48 h post-transfection and were used to detect the changes of p38 MAPK mRNA expression.

### Luciferase Assay

We constructed the pMIR-report-3′UTR plasmid for the p38 MAPK gene. The pMIR-report vector was purchased from Addgene (Cambridge, MA). The 3′UTR sequence (1608 bp) from rat p38 MAPK mRNA were isolated by qPCR with the primers containing restriction enzymes: F: 5′-GACTAGTCGGGTGCATGTATGTGCGTAG-3′; R: 5′-CAAGCTTGTGGAGCTGGACTGCATACTG-3′. The PCR fragments were digested and cloned into the pMIR-Report vector at the SpeI and HindIII sites. In addition, corresponding to the wild-type 3′UTR reporter plasmid (pMIR-p38 MAPK 3′UTR-wt), we produced mutant-type plasmid (pMIR-p38 MAPK 3′UTR-mt) using the QuikChange Kit (Life Technology, CA), in which specific miRNA binding sites were mutated with the following primers: 5'-GCAAGGGTTCAGGTGCGCTGGGCGTTCCGTTTCCTGCACCAC-3'; 5'-GTGGTGCAGGAAACGGAACGCCCAGCGCACCTGAACCCTTGC-3'.

ND8/34 cells were seeded onto 24-well plates the day prior to transfection. They were transfected with 0.5 µg pMIR-p38 MAPK 3′UTR-wt or pMIR-p38 MAPK 3′UTR-mt and 50 nM miR-125a-3p mimic, inhibitor or non-target control (Life Technology, NY, USA), and 0.05 µg of the Renilla luciferase vector pRL-TK (Promega, CA, USA) for normalization. Luciferase activity was measured at 48 h post-transfection using a dual-luciferase assay kit (Promega, Madison, USA). Firefly luciferase activity was normalized to Renilla luciferase activity. All transfection experiments were conducted in triplicate.

### Statistical Analysis

Statistical analysis was performed using GraphPad software (GraphPad software, San Diego, CA, USA). Data are presented as the mean ± standard deviation (SD). An independent-samples t-test or ANOVA (one-way analysis of variance) was used to determine the difference between or among groups. Pearson correlation analysis was used to analyze the correlations of miR-125a-3p expression with p38 MAPK, CGRP mRNA expression and head withdrawal threshold. *p*<0.05 was considered statistically significant.

## Results

### Behavioral Tests

Head withdrawal thresholds before injection were not significantly different in all rats. The values (121.5±6.59 g) 3 days after normal saline injection did not change significantly compared with those before injection (123.0±5.96 g). Whereas 3 days after CFA injection, the values (80.25±7.35 g) decreased significantly compared with those before CFA (131.0±7.23 g) or after normal saline injection (*p*<0.05). These data indicated that CFA injection induced a valid pain in orofacial regions.

### MiRNA Expression Profile with miRNA Microarray

**Figure 1:**
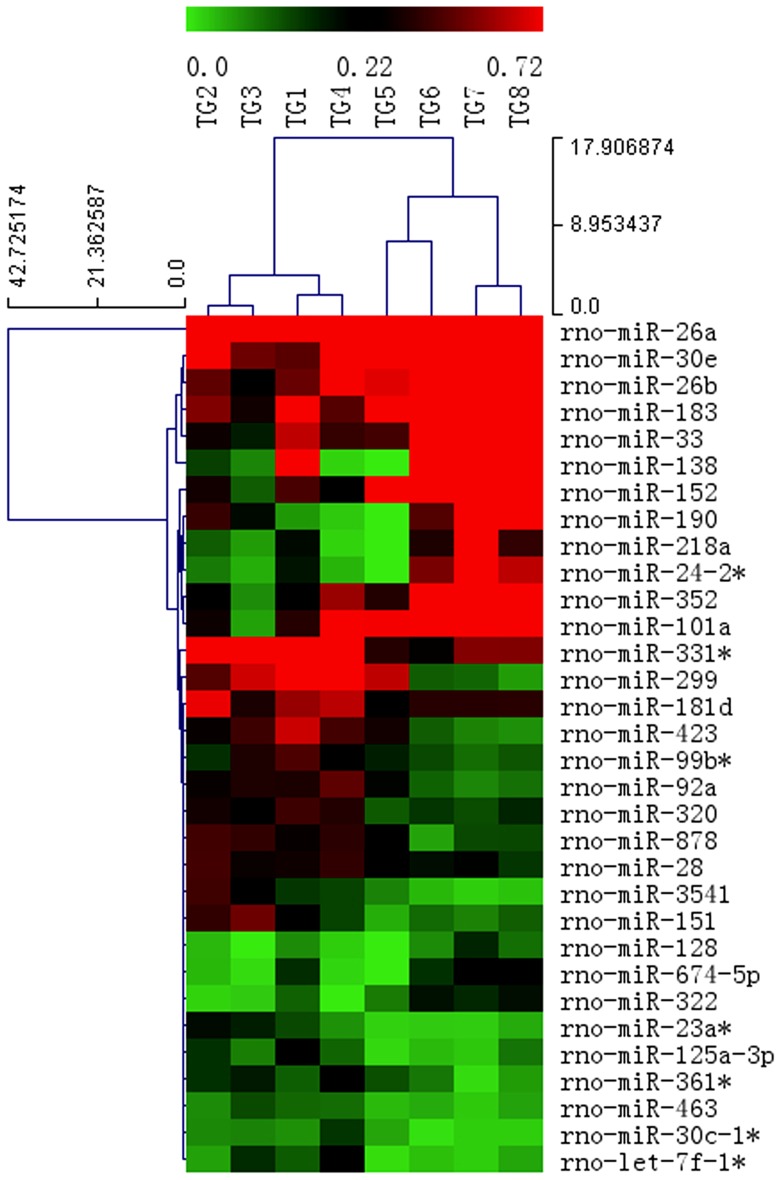
Hierarchical cluster analysis of differentially expressed miRNAs in injection-ipsilateral TGs. TG1, TG2, TG3 and TG4 were from rats with facial normal saline injection, whereas TG5, TG6, TG7 and TG8 were from rats with facial CFA injection (n = 4/group). Each row represents a miRNA, and each column represents a TG sample. Red represents high expression, and green represents low expression (fold change >1.5).

The present miRNA microarray contained 2056 miRNA probes among which 890 rat miRNAs were included. Expression profile in the ipsilateral TGs of rats following normal saline- or CFA-injection into orofacial skin was detected. After normalization, the average value for each miRNA spot was used for statistics. Based on the fold change ≥ 1.5, *p*-value ≤0.05 and FDR ≤0.05, 32 miRNAs were differentially expressed in ipsilateral TGs of CFA-injected rats, among which 12 were up-regulated and 20 were down-regulated. The miRNA differential expressions in CFA-injected rats increased by 2.1- to 13.7-fold or decreased by 2.0- to 11.6-fold compared with those in normal saline-injected rats. Based on the differential expressions of miRNAs, a hierarchical clustering analysis was conducted (Figure 1).

### Validation of Microarray Results using qPCR

**Figure 2:**
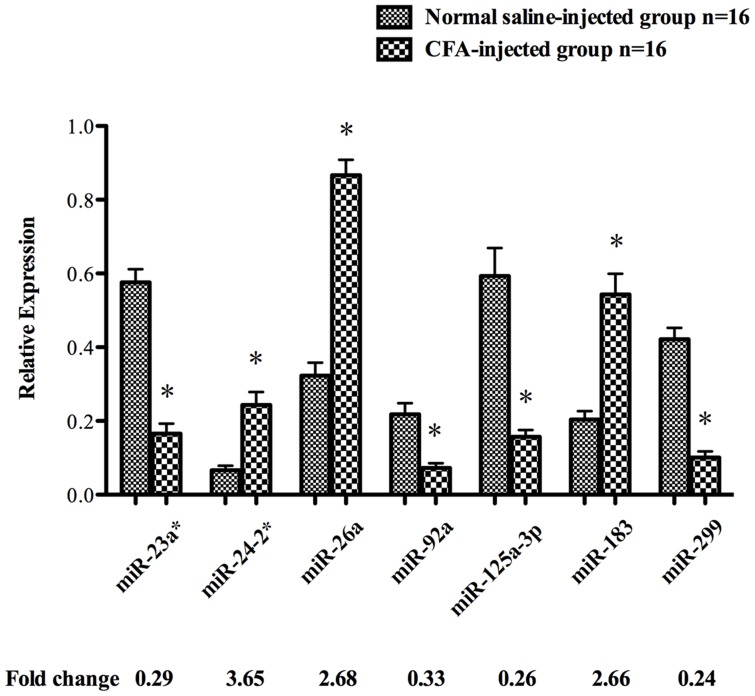
Differential expression of miRNAs detected using qPCR. The bar graphs show the expression of miR-23a*, -24-2*, -26a, -92a, -125a-3p, -183 and -299 in injection-ipsilateral TGs of rats 3 days following facial normal saline injection and CFA injection (n = 16/group). The results show the relative expression following normalization. Values are the means ± SD. *p<0.01.

The differentially expressed miRNAs were selected and further validated by qPCR and the result showed the same expression tendency as microarray analysis although the discordance was not identical. The expressions of miR-24-2*, -26a, and -183 were up-regulated, whereas those of miR-23a*, -92a, -125a-3p and -299 were down-regulated in CFA-injected compared with normal saline-injected samples (*p*<0.01) (Figure 2).

### MiRNA Target Genes Prediction and GO Analysis

The target genes of miR-23a*, -24-2*, -26a, -92a, -125a-3p, -183, and -299 were predicted by miRWalk and other programs based on the mRNA 3' UTR region. GO analysis indicated these predicted target genes were involved in many common biological processes, which were related to the regulation of response to stimulus, multicellular organismal development process, regulation of signaling, nervous system development, etc. Moreover, they were also related to some specific biological process categories involving pain or inflammation. For example, the predicted target genes of miR-125a-3p were found to be involved in some biological process including transmission of nerve impulse, regulation of action potential in neuron, regulation of inflammatory response, regulation of gene expression, synapse assembly, synaptic transmission, long-term synaptic potentiation, regulation of membrane potential, neuron fate commitment (Table 2).

**Table 2:** GO analysis showing specific biological process categories related to pain or inflammation where the target genes were involved.

MiRNAs	Specific biological process category related to pain or inflammation
miR-23a*	Neurotransmitter transport, neuromuscular synaptic transmission, transmission of nerve impulse, synaptic transmission, synaptic vesicle transport, ensheathment of neurons, positive regulation of immune system process, sensory perception of pain
miR-24-2*	Inflammatory response, cellular response to calcium ion, response to virus, immune system process, cellular response to stimulus, ventral spinal cord interneuron differentiation
**miR-26a**	Positive regulation of MAP kinase activity, glial cell apoptosis, positive regulation of dendrite morphogenesis, neuron development, neuron projection development, midbrain development, neuron projection regeneration
**miR-92a**	Neuron projection regeneration, ventral spinal cord development, ventral spinal cord interneuron differentiation, cell differentiation in hindbrain
**miR-125a-3p**	Transmission of nerve impulse, regulation of action potential, regulation of inflammatory response, regulation of gene expression, synapse assembly, synaptic transmission, long-term synaptic potentiation, regulation of action potential in neuron, regulation of membrane potential, neuron fate commitment
**miR-183**	Response to oxidative stress, cellular potassium ion transport, potassium ion transmembrane transport, neuromuscular process controlling balance, response to stress
**miR-299**	Neuron projection morphogenesis and development, regulation of inflammatory response, regulation of gene expression

### Correlation of miR-125a-3p Expression with the Head Withdrawal Threshold, p38 MAPK and CGRP mRNA Expression in Rat TGs

Based on GO analysis, the predicted target genes of miR-125a-3p including p38 MAPK, Cox6c and NMD3A are associated with inflammation and pain process. We detected the level of p38 MAPK level at different time points and analyzed the correlation of miR-125a-3p level with p38 MAPK, CGRP mRNA expression and the head withdrawal threshold.

**Figure 3:**
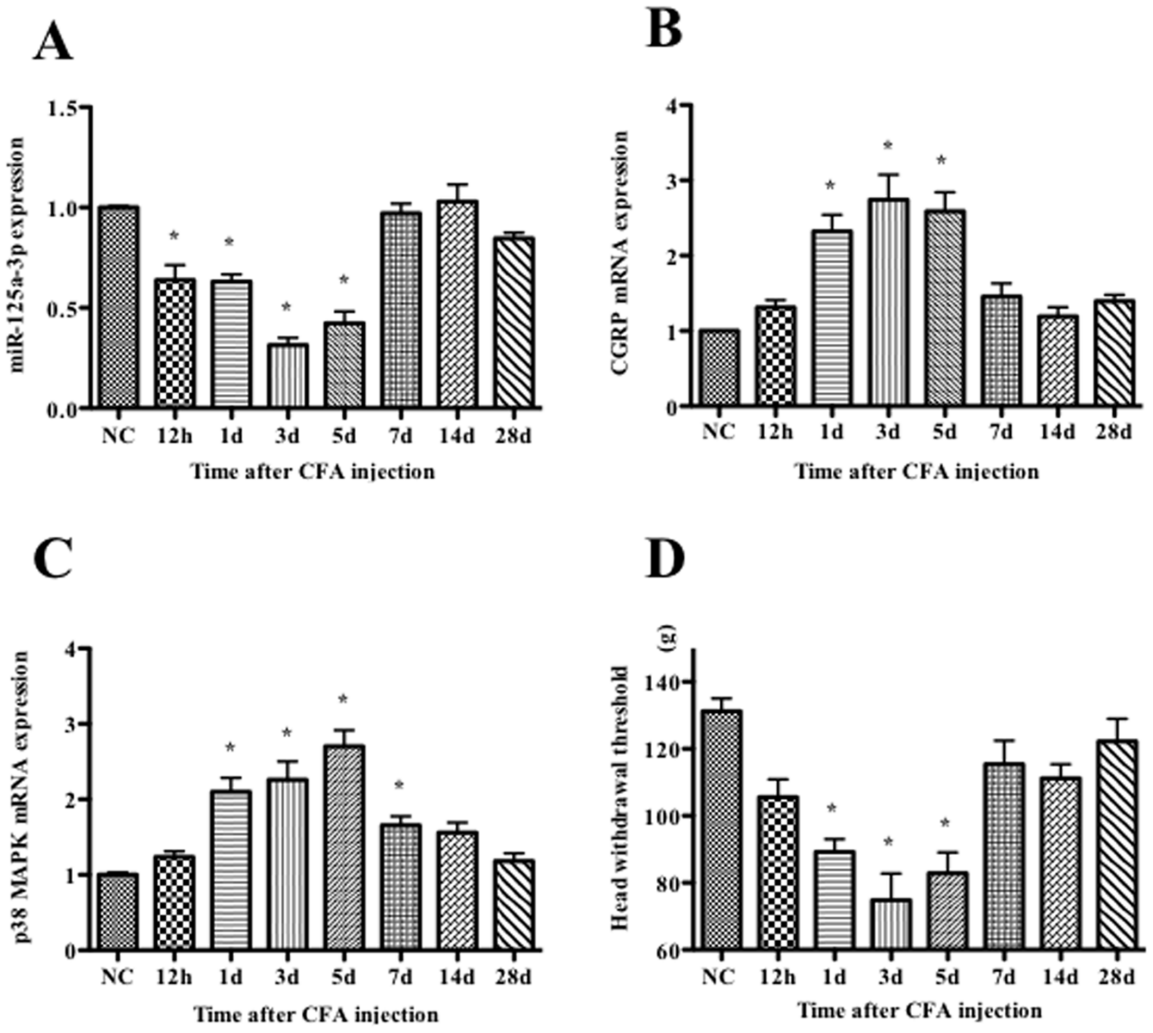
Expression of miR-125a-3p (A), CGRP (B) and p38 MAPK (C) mRNA in ipsilateral TGs and head withdrawal thresholds (D) in ipsilateral orofacial region at different time points following CFA injection into rat orofacial skin. The results are shown as a percentage compared with the NC (normal control, baseline before injection). Values are the means ± SD (n = 5/time point). ^*^ *p*<0.01.

Compared with the baseline of normal control, the expression of miR-125a-3p after CFA injection decreased significantly from 12 h, attained the lowest level at 3 d, and then began to rise gradually at 5 d, but the decrease was still significant (*p*<0.01). At 7 d, the miR-125a-3p level tended toward the baseline, and then returned to the baseline at 14 d (Figure 3A).

CGRP and p38 MAPK mRNA expression were both significantly increased from 1 d and reached their highest levels at 3 d for CGRP and 5 d for p38 MAPK, respectively, following CFA injection (*p*<0.01). The CGRP expression level gradually returned to the baseline at 14 d, whereas the p38 MAPK level gradually returned at 28 d after CFA injection (Figure 3B and Figure 3C).

Compared with normal controls (131.2±3.81 g), the head withdrawal thresholds of the CFA injection group were significantly decreased from 1 d (89.2±3.83 g) to 5 d (82.8±6.27 g) with the lowest value at 3 d (74.8±7.97 g) (*p*<0.01) (Figure 3D). The Pearson correlation analysis indicated that miR-125a-3p expression level was positively correlated with the head withdrawal threshold (*R* = 0.731, *p*<0.001) and negatively related to p38 MAPK and CGRP mRNA level (*R* = −0.673 and −0.699, respectively, *p*<0.001).

### MiR-125a-3p negatively regulated the p38 MAPK expression in ND8/34 cells

**Figure 4:**
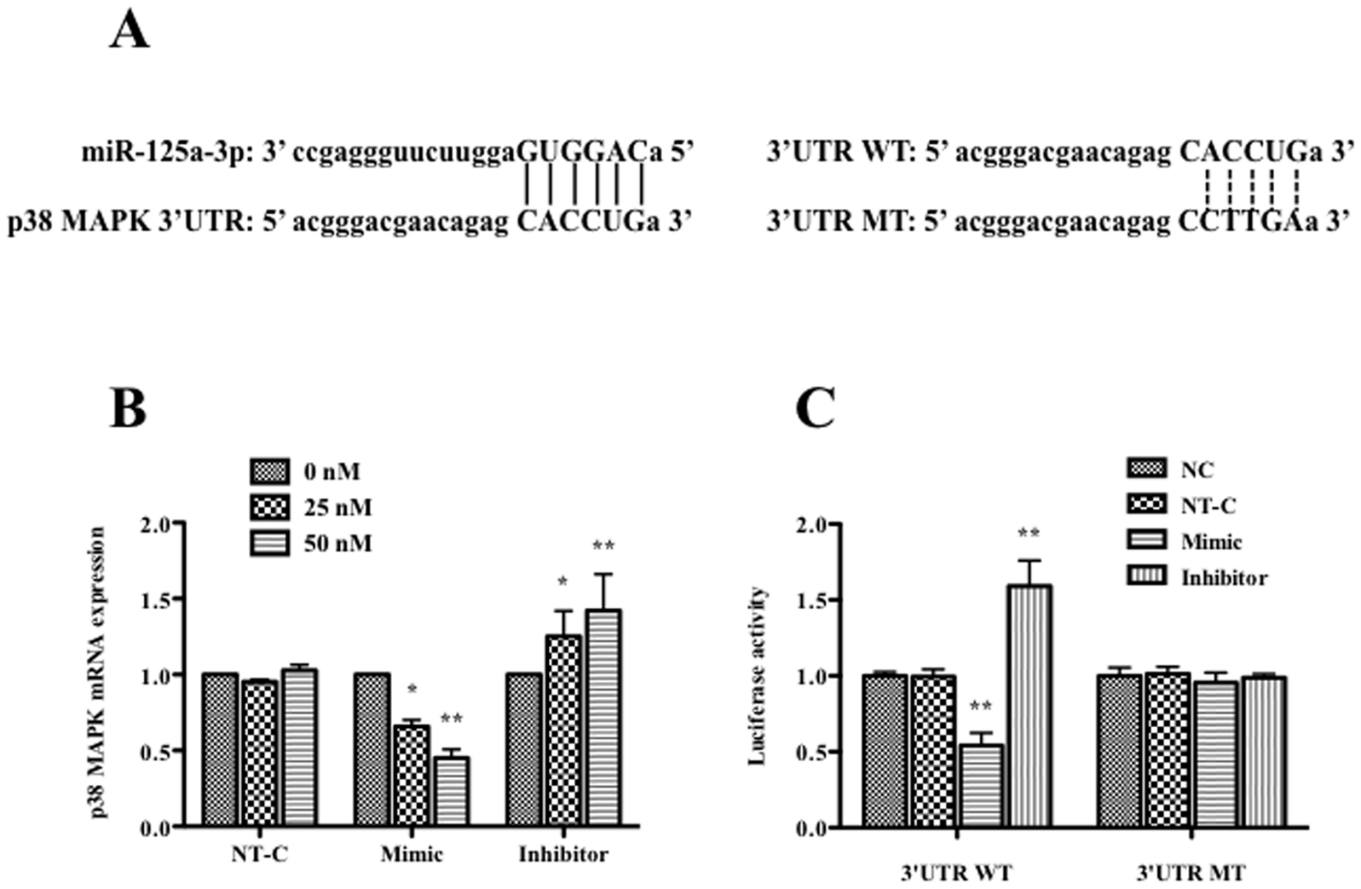
Effects of miR-125a-3p on p38 MAPK expression and luciferase activity in ND8/34 cells. (A) Binding sites alignment of the mature miR-125a-3p and the 3′UTR of rat p38 MAPK gene (left) and base pair difference between wild type (WT) and mutant type (MT) of p38 PAPK gene 3′ UTR (right). (B) The expression of p38 MAPK mRNA due to the transfection of 0, 25 or 50 nM miR-125a-3p mimic, inhibitor or non-target control (NT-C) in ND8/34 cells detected by qPCR. (C) The luciferase activity of p38 MAPK 3′UTR WT or 3′UTR MT due to the transfection of 50 nM miR-125a-3p mimic, inhibitor or NT-C in ND8/34 cells. * *p*<0.05, and ** *p*<0.01.

In order to determine whether miR-125a-3p plays a role in regulating p38 MAPK expression, we first aligned the miR-125a-3p sequence with p38 MAPK 3′UTR on the website (http://www.umm.uni-heidelberg.de/apps/zmf/mirwalk/), and found there were 6 base pairs of binding sites matched (Figure 4A). Then we detected the p38 MAPK mRNA level in ND8/34 cells after miR-125a-3p transfection. p38 MAPK mRNA levels after the transfection of miR-125a-3p non-target control with different doses (0, 25, 50 nM) had no significant changes. Compared with non-target control, the expression of p38 MAPK mRNA was significantly decreased at 48 h after miR-125a-3p mimic transfection (*p*<0.01). The inhibition of miR-125a-3p on p38 MAPK mRNA was dose-dependent. Conversely, p38 MAPK mRNA expression was significantly increased in a dose-dependent manner when miR-125a-3p inhibitor was transfected into ND8/34 cells compared with the non-target control (*p*<0.01) (Figure 4B).

Finally, we detected whether p38 MAPK was the direct target of miR-125a-3p using luciferase assay. We constructed the reporter plasmids containing 3′-UTR of p38 MAPK gene, as well as the mutant constructs with the potentially mutated miR-125a-3p binding sites. These plasmids were co-transected with miR-125a-3p mimic or inhibitor into ND8/34 cells, and the transcriptional activity of p38 MAPK was evaluated by luciferase assay. The results showed that overexpression of miR-125a-3p significantly diminished luciferase activity (decrease ∼35% at 25 nM, and ∼55% at 50 nM of miR-125a-3p mimic, respectively, *p*<0.01), while the miR-125a-3p inhibitor increased the luciferase activity (increase ∼20% at 25 nM, and ∼40% at 50 nM of miR-125a-3p, *p*<0.05 and *p*<0.01, respectively), both in a dose-dependent manner. However, luciferase activity had no significant changes when the ND8/34 cells were transfected with the mutant plasmids. These results indicate that p38 MAPK gene is directly regulated by miR-125a-3p (Figure 4C).

## Discussion

The present study screened the differential miRNAs using a miRNA microarray in the ipsilateral TGs of rats with CFA-induced orofacial inflammatory pain. In 890 rat mature miRNAs, 32 miRNAs were differentially expressed compared with the control rats. The results of the miRNA microarray were further validated by qPCR, and both methods showed the same expression tendency. Furthermore, we focused on a down-regulated miRNA, miR-125a-3p, and found a negative relationship between miR-125a-3p and p38 MAPK expression. Luciferase assay indicated p38 MAPK is the direct target of miR-125a-3p. This is the first time to detect the miRNA profile and to explore the role of miR-125a-3 in TGs following orofacial pain.

Many miRNAs were differentially expressed in the injured rat dorsal root ganglion (DRG) neurons or spinal cord following neuropathic pain induced by spinal nerve injury, such as miR-21, -96, -182, -183, -103, -1, -16, -206, -103, 221, -500 and -551b [10], [15], [28]–[30]. However, only a few miRNAs was reported in orofacial pain. Bai *et al*. [16] first described mature miR-10a, -29a, -98, -99a, -124a, -134 and -183 expressions in the TGs of rats with inflammatory muscle pain induced by facial CFA injection. Another study indicated that miR-155 and -223 increased significantly in mice prefrontal cortex after inflammatory pain induced by facial carrageenan injection [14]. The present study detected the differential expression of miR-23a*, -24-2*, -26a, -92a, -125a-3p, -183 and -299 in the TGs after orofacial inflammatory pain induced by CFA. However, some miRNA expressions in our study were different from those in previous studies. Our results indicated that miR-183 expression was significantly up-regulated at 3 d following CFA injection, whereas in the ipsilateral mandibular division of the TG, the level of miR-183 expression was reduced significantly within 4 h and then recovered to a normal level at 4 d after CFA injection into the rat masseter muscle [16]. In addition, spinal nerve ligation causes down-regulation of miR-183 at 2 weeks in the ipsilateral compared with the contralateral DRG [10]. The differential expression way was also observed in miR-125a. Some studies showed increased miR-125a expression [18], [19], [31] and whereas others did not [17]. The present study indicated a decreased miR-125a-3p expression. The inconsistent expression pattern might be due to the difference of temporal-spatial expression and pain types as well as the function complexity of miRNAs under different painful or stressful conditions. The same experiment animal species, types of pains, time points selected and technique methods will help to detect the miRNAs expression and illustrate the molecular mechanism of pain.

It has been speculated that miRNAs may participate in controlling different states of pain by regulating the expression of pain-relevant target genes [29]. Previous reports have indicated miR-103 and miR-1a-3p modulate neuropathic or cancer pain through Cav1.2 calcium channel and clcn3, respectively [11], [28]. In the present study, the predicted target genes of miR-23a*, -24-2*, -26a, -92a, -125a-3p, -183, and -299, including NMDA, MAPK and Tac were found to be involved in neuropathic, inflammatory pain, or other states of pain [26], [32]–[34]. Furthermore, GO analysis indicated miR-125a-3p was involved in some processes of pain transduction including transmission of nerve impulse, synaptic transmission, regulation of action potential in neuron, regulation of inflammatory response and regulation of gene expression, which reminds us to pay more attention to it.

As we know, miR-125 has been considered as a biomarker of many cancers, such as gastric cancer and non-small cell lung cancer [35], [36]. Recently, emerging evidence showed miR-125 is involved in the regulation of cell stress, inflammation and pain. Murata *et al*. [18] detected the increased plasma concentration of miR-125a-5p and suggested miR-125a-5p may be a potential diagnostic marker of rheumatoid arthritis. Kumar *et al*. [17] described the down-regulation of miR-125a-3p expression was involved in West Nile virus-induced neuroinflammation in mice brain by regulating some anti-viral cytokines.

Other studies had focused on the relationship between miR-125 and p38 MAPK. Ultraviolet radiation stress induced the up-regulation of miR-125b, which promoted cell survival by negatively regulating p38α expression through targeting its 3′-UTR [19]. Candida albicans and lipopolysaccharide (LPS)-induced inflammation up-regulated miR-125a expression in macrophages and the pri-miR-125a transcription was also regulated by MAPK signal pathway [31].

In the present study, CFA injection into orofacial skins resulted in the high expression of CGRP gene and mechanical hypersensitivity, so CFA injection produced a reliable pain model. The pain induced a decreased miR-125a-3p expression and increased p38 MAPK expression in vivo. Previous study showed orofacial pain induced by occlusal interference was associated with the activation of p38 MAPK pathway [26]. Our result also showed that down-regulation of miR-125a-3p significantly increased MAPK mRNA level in vitro, and luciferase assay confirmed a direct binding of miR-125a-3p to the 3′UTR of p38 MAPK mRNA. The results indicated miR-125a-3p might be negatively correlated to the severity of inflammation and pain in orofacial region and might participate in the process of the development of inflammatory pain following CFA injection via the p38 MAPK signal pathways.

## Conclusions

To the best of our knowledge, this report describes for the first time a systematic analysis of the expression profile of miRNAs performed on the ipsilateral TG of an orofacial inflammatory pain animal model. Besides, our study also indicates that miR-125a-3p might have a negative correlation with the development and maintenance of inflammatory pain via regulating the p38 MAPK signaling.
